# Ab initio study of the structure, elastic, and electronic properties of Ti_3_(Al_1−n_Si_n_)C_2_ layered ternary compounds

**DOI:** 10.1038/s41598-021-84466-5

**Published:** 2021-03-02

**Authors:** S. T. Ahams, A. Shaari, R. Ahmed, N. F. Abdul Pattah, M. C. Idris, B. U. Haq

**Affiliations:** 1 Department of Physics, Faculty of Science, Universiti Teknologi Malaysia, UTM Skudai, 81310 Johor Bahru, Johor Malaysia; 2 Department of Pure and Applied Physics, Faculty of Science, Adamawa State University, Mubi, Nigeria; 3 Center for High Energy Physics, University of the Punjab, Quaid-E-Azam Campus, Lahore, 54590 Pakistan; 4 Department of Physics, Sule Lamido University, Kafin Hausa, Jigawa State Nigeria; 5 Advanced Functional Materials and Optoelectronics Laboratory (AFMOL), Department of Physics, Faculty of Science, King Khalid University, P.O. Box 9004, Abha, Saudi Arabia

**Keywords:** Materials science, Physics

## Abstract

The MAX phase materials such as layered ternary carbides that simultaneously exhibit characteristics of metallic and ceramic materials have received substantial interest in recent years. Here, we present a systematic investigation of the electronic, structural stabilities, and elastic properties of Ti_3_(Al_1−n_Si_n_)C_2_ (n = 0,1) MAX phase materials using the ab initio method via a plane-wave pseudopotential approach within generalized-gradient-approximations. The computed electronic band structures and projected density of states show that both Ti_3_SiC_2_ and Ti_3_AlC_2_ are metallic materials with a high density of states at the Fermi level emanating mainly from Ti-3d. Using the calculated elastic constants, the mechanical stability of the compounds was confirmed following the Born stability criteria for hexagonal structures. The Cauchy pressure and the Pugh’s ratio values establish the brittle nature of the Ti_3_SiC_2_ and Ti_3_AlC_2_ MAX phase materials. Due to their intriguing physical properties, these materials are expected to be suitable for applications such as thermal shock refractories and electrical contact coatings.

## Introduction

MAX phases are a family of over 70 synthesized ternary nitrides and carbides of general stoichiometry M_*n*+1_AX_*n*_ where *n* = 1, 2, or 3, M denotes early transition metals (TM), A represents A- group elements (mostly from group IIIA or IVA) and X is either nitrogen (N) or carbon (C)^1–4^. Resulting from their general formula, different groups of MAX phases are characterized as 211, 312, 413, 614, and so on^5^. Some MAX phases were discovered experimentally by Nowotny et al*.* about forty years ago^6^. In the early 1960s, the majority of MAX phases were discovered in a succession of experiments by Nowotny and his co-workers^7^. However, these discovered MAX phases did not receive adequate interest till the Barsoum and El-Raghy synthesized and fully characterized the bulk Ti_3_SiC_2_ MAX phase in 1996^8^. Thereafter, interest in the layered ternary compounds increased rapidly^9–11^. Based on the web of science (WOS)^12^, to date, there are over 4,168 published papers on MAX compounds alone, with Ti_3_SiC_2_ having roughly half of the published works in the past six years^13,14^. MAX phase family is a large group of layered ternary carbides and nitrides that crystalizes into the hexagonal structure of spacegroup No. P_63_/mmc. Withthe characteristics of metallic as well as ceramics materials^15^, where each group member contains at least two forms of ionic, covalent, or metallic chemical bonds^16^. The MAX phases such as Ti_3_SiC_2_ and Ti_3_AlC_2_ are a 312 class of layered ternaries where the individual phases differ by the number of M-layers parting the A-layers in the 312-MAX phases^17–21^. These compounds combine some characteristics of metals like strong compressive strength, high fracturing strength, hardness, ductility, good electrical and thermal conductivity, high stiffness, damage tolerance, relatively low thermal expansion coefficient. Like ceramics they have outstanding thermal and chemical tolerance. . Furthermore, these compounds are considered as one of the best classes of materials for coating on steel surfaces in heavy liquid metals and as pump impellers. However, Ti_3_SiC_2_ and Ti_3_AlC_2_ are among the best-accepted representatives of the MAX phase compounds and are known as the best thermal conductors than titanium metal^22–26^.

First-principles approaches are widely employed to study the properties of MAX phases, for example, M_2_GaN (M = Ti, V and Cr)^27^, Ti_2_TlC, Zr_2_TlC, and Hf_2_TlC^17,23^, Ti_3_AlC_2_ and Ti_2_SiC_2_^20^. Zhou et al.^28^ reported the distribution of charge density on the (1120) plane of Ti_3_AlC_2_, where robust directional Ti-C-Ti-C-Ti covalent bond chains were observed that linked to fairly weaker Ti–Al covalent bindings. In a similar study of electronic structure and bonding properties of Ti_3_AlC_2_, Wang and Zhou^29^ reported that the electrical conductivity of Ti_3_AlC_2_ decreases with increasing pressure, and over the whole pressure range, the material was found to exhibit elastic anisotropy. Son et al*.*^30^ have used density functional theory (DFT) to analyze the structural, elastic, and thermodynamic properties of Ti_3_SiC_2_ and Ti_3_AlC_2_ crystals. In order to discover the finite-temperature properties of these crystals, the vibrational, mechanical, quasi-harmonic contributions, and anharmonic adjustment to the total free energy of the systems were determined and extrapolated and the functions of electron localization, charge densities, electronic and vibrational densities have been studied.

Zhou and Zhimei investigated the electronic structure and chemical bonding in layered machinable Ti_3_SiC_2_^31^. According to them, bonding within Ti_3_SiC_2_ is facilitated by metallic, covalent, and ionic bonding due to the strong Ti-C-Ti-C-Ti covalent bond strings in the structure^31^. In recent years, several studies have been carried out on the mechanical properties, and structural stabilities of Ti_3_SiC_2_ and Ti_3_AlC_2_^32–34^ that reported their excellent structural properties that are suitable for many practical applications. Synchrotron x-ray diffraction measurements showed that Ti_3_SiC_2_ and Ti_3_AlC_2_ are stable materials under pressure from 0 to 61 GPa at room temperature^35^. Thermal stability of bulk Ti_3_AlC_2_ has been investigated^36^ within 1100–1400 °C, and hydrogen has been found to alter the properties and stability of the MAX phase. Analogous facts have also been noticed in the temperature range 1473–1673 K in bulk Ti_3_SiC_2_ in the hydrogen atmosphere and it was found that the dissociation of Ti_3_SiC_2_ was accelerated by hydrogen^37^.

Herein, we have investigated Ti_3_SiC_2_ and Ti_3_AlC_2_ using plane-wave pseudopotentials (PW-PP) approach in the framework of DFT. Since hardness varies from one material to another as commonly acknowledged by materials scientists, materials with Vickers hardness greater than 40 GPa are categorized as superhard^38,39^. We have achieved a result which by far characterizes Ti_3_SiC_2_ and Ti_3_AlC_2_ as superhard materials which we feel none of the studies conducted so far could address.

## Result and discussion

### Structural properties

**Figure 1:**
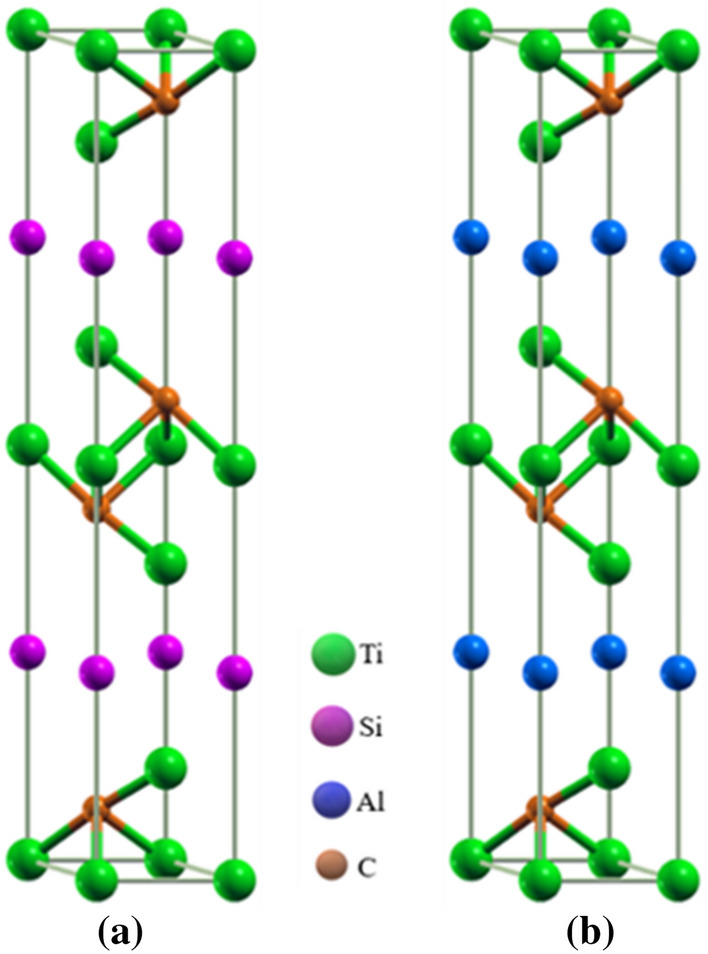
Crystal structure of (**a**) Ti_3_AlC_2_, (**b**) Ti_3_SiC_2_ MAX phase compounds.

The layered ternary Ti_3_(Al_1−n_Si_n_)C_2_ (n = 0, 1) compounds are based on the layers of hexagonally close-packed Si/Al and Ti layers with C occupying octahedral centers between the Ti layers as depicted in Fig. 1. The end phases could also be characterized as alternating stacking of two layers of a planar close-packed Si/Al and Ti_6_C octahedral layers. The Ti atom is found to be located at 4f. (0.33, 0.67, *z*), Al/Si atoms are positioned at 2b (0, 0, 0.25) whereas the atom of C is at 4f. (0.33, 0.67, *z*) Wyckoff positions. Figure 1 illustrates the crystal symmetries of the studied compounds and their computed structural parameters as well as the experimental results from available literature(s) are summarized in Table 1. The results of the equilibrium lattice constants, bulk modulus, and its pressure derivative are computed by fitting the obtained data of the equilibrium energy as well as volume to the second-order Birch-Murnaghan’s equation of state (EOS)^40^. The obtained results showed the reasonability of our calculations.

**Table 1:** Calculated equilibrium lattice parameters *a, c*, *c/a* ratio, volume, *V*, bulk modulus *B*_*o*_, and its pressure derivative${, B}_o^{\mathrm{^{\prime}}}$ and values from the literature.

Compound	Reference	*a* (Å)	*c* (Å)	*c/a*	*V* (Å^3^)	*B*_*o*_ (GPa)	${B}_o^{^{\prime}}$
Ti_3_AlC_2_	This work	3.0781	18.7681	6.0973	153.93	145	2.81
	Exp.^41^	3.082	18.642	6.0487			
	Calc.^30^	3.083	18.652	6.040		163.35	
Ti_3_SiC_2_	This work	3.0697	17.6864	5.7000	145.60	180.5	4.14
	Exp.^41^	3.075	17.734	5.7672			
	Calc.^30^	3.077	17.715	5.7572		192.61	

1 $$ ~E\left( V \right) = E_{o} \frac{9}{{16}}B_{o} \left[ {\left( {4 - B_{o}^{'} } \right)\frac{{V_{o}^{3} }}{{V^{2} }} - \left( {14 - 3B_{o}^{'} } \right)\frac{{V_{o}^{{7/3}} }}{{V^{{4/3}} }}\left( {16 - 3B_{o}^{'} } \right)\frac{{V_{o}^{{5/3}} }}{{V^{{2/3}} }}} \right] $$

One can easily note that the difference between our obtained results and experimental data of equilibrium lattice parameters is less than 1%, showing that our results obtained at the level of the Perdew-Burke-Ernzerhof (PBE) type of generalized gradient approximations functional are sufficiently reliable. In Table 1, the bulk modulus of Ti_3_SiC_2_ is higher than that of Ti_3_AlC_2_, showing that Ti_3_SiC_2_ is harder than Ti_3_AlC_2_.

### Electronic properties

**Figure 2:**
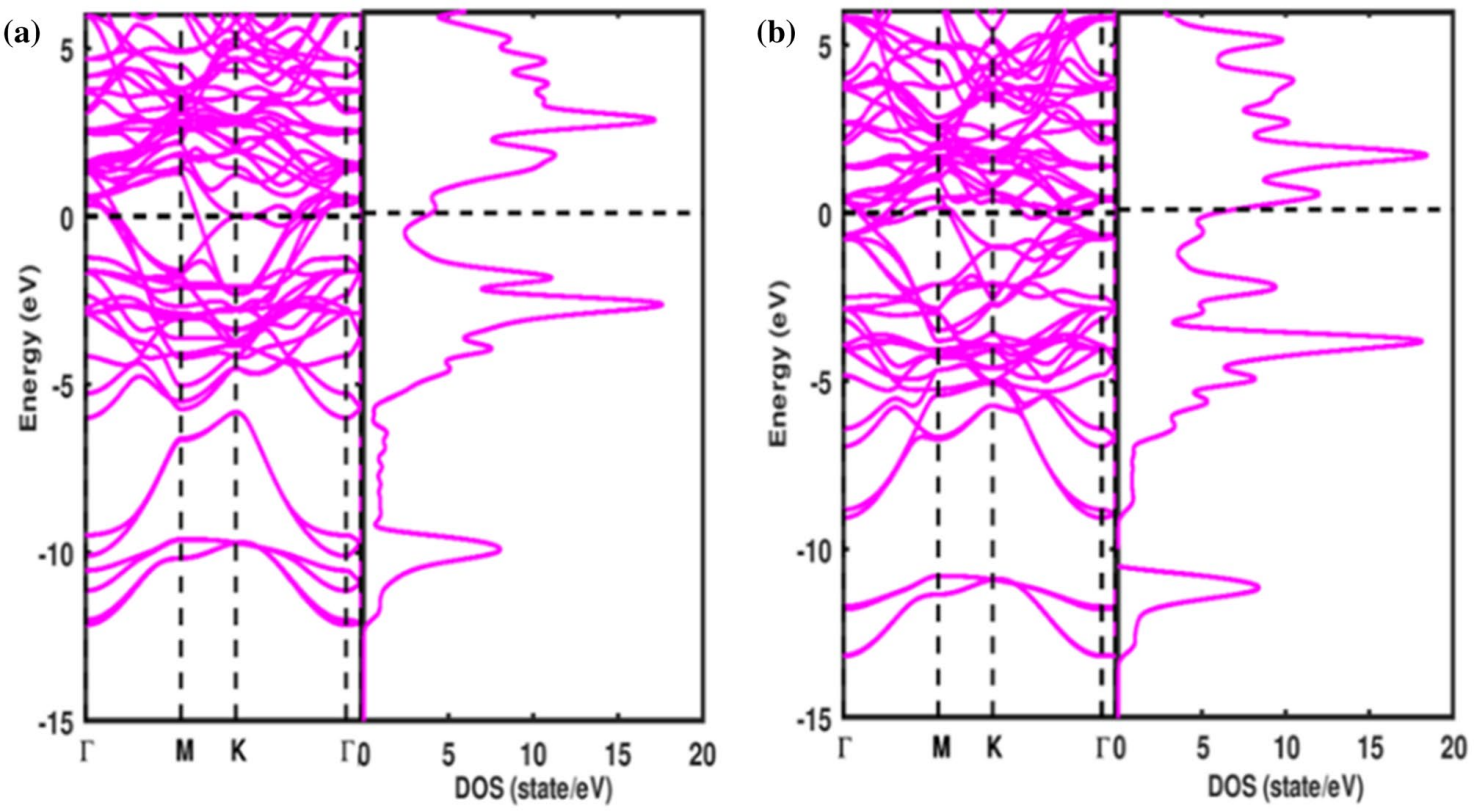
Band structures and TDOS of (**a**) Ti_3_SiC_2_, (**b**) Ti_3_AlC_2_ MAX phase compounds.

Figure 2 demonstrates the band structures and total density of states (TDOS) computed along the high symmetry points in the brilluoin zone (BZ) using the equilibrium lattice parameters. It is seen that both valence bands and conduction bands overlap significantly resulting in no energy gap at the Fermi level. Thus, the studied compounds demonstrate metallic character which is a common feature of the MAX phase materials. However, there are more valence electrons in the Ti_3_SiC_2_ unit cell than in Ti_3_AlC_2_. This gives rise to the further occupation of the bonding states near the Fermi level. The substitution of Si by Al in Ti_3_AlC_2_ presents additional valence electrons per atom, and consequently, the Fermi level is moved to a higher energy level. This suggests that the increased extra valence electrons fill in the Si/Al-Ti *p-d* hybridized bonding states as well as the metal to metal *d-d* consequential bonding.

Accordingly, the filling of the bonding orbitals rises the strength of the bond and thereby increasing the bulk moduli. The energy band also exhibits a highly anisotropic character along with lesser *c*-axis energy dispersion. The anisotropy of the band structure near and below the Fermi level implies that, for single crystals, both Ti_3_SiC_2_ and Ti_3_AlC_2_ are conductors and anisotropic, and electrical conductivity is lowered along *c* direction than the *ab*- plane similar to the observed trend in the literature^28^.

**Figure 3:**
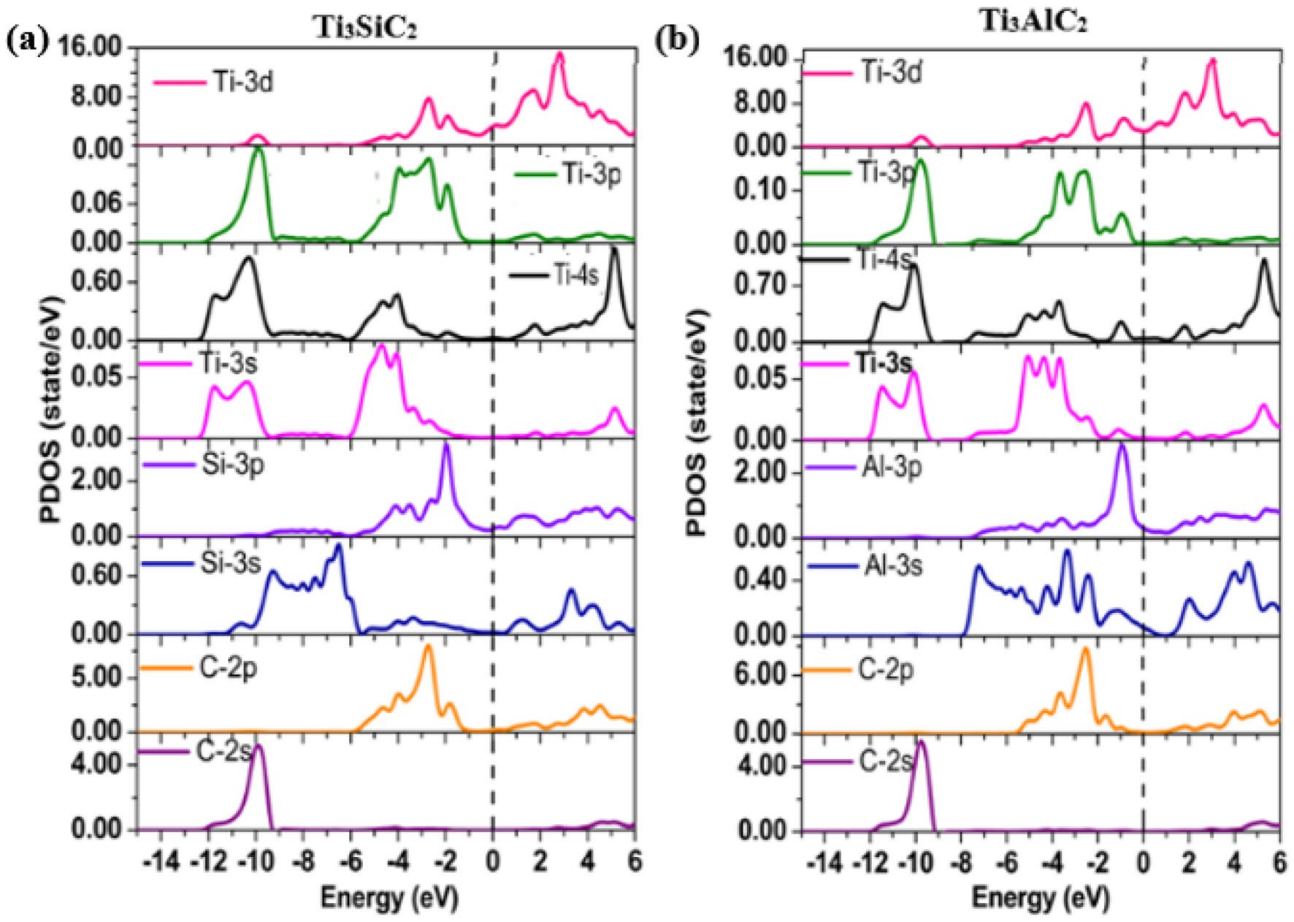
Calculated PDOS of (**a**) Ti_3_SiC_2_ and (**b**) Ti_3_AlC_2_ MAX phases.

The investigated total densities of states (TDOS) plot for Ti_3_SiC_2_ and Ti_3_AlC_2_ presented in Fig. 2 points out that the peak structures and corresponding heights of the peaks are equivalent, signifying resemblance in chemical bonding. The TDOS per unit cell at the Fermi level for Ti_3_SiC_2_ and Ti_3_AlC_2_ are 4.029 and 6.855 states/eV, respectively. Therefore, there is an increasing trend in the DOS at the Fermi level with an increasing number of valence electrons of the transition metals showing that the transition metal bands play a dominant role in the TDOS and their electrical transport properties. Analysis of bonding properties is obtained from the PDOS of each contributing element in Fig. 3. Here, the width of Al-3 s and Si-3 s states are wider for each one than that of the C-2 s state. With several less contributing peaks in the Al/Si-are due to 3 s states. The Al/Si-3 s energy states show that there are *s-p* interactions in Al/Si, i.e. close-packed layer of Al/Si atoms are bonded through *s-p* interactions. For the energy range -12 eV to -9.4 eV in the valence bands of both Ti_3_SiC_2_ and Ti_3_AlC_2_, there is a high degree of hybridization of C-2p with Ti-3d states, which suggests covalent bonding between them. Hence the chemical stability is largely attributed to the *p-d* hybridization. Therefore, the Ti-3d and C-2p hybridization is a driving bonding force in Ti_3_SiC_2_ and Ti_3_AlC_2_, similar to bonding properties in some 312 MAX phases like Ti_3_SnC_2_ and Ti_3_GeC_2_^42,43^.

### Elastic properties

Investigations of elastic constants are vital for applications related to the mechanical properties of solids. They provide information on stability, bonding, ductility, brittleness, anisotropy, compressibility, Vicker’s hardness, and stiffness of solids^44,45^. For hexagonal crystals structures, five independent elastic constants (${C}_{11}$, ${C}_{12}$, ${C}_{13}$,${C}_{33}$, ${C}_{44}$) are required. Table 2 summarizes our computed results of the five independent elastic constants of Ti_3_SiC_2_ and Ti_3_AlC_2_ alongside available experimental and theoretical data.

**Table 2:** Computed elastic constants C_ij_ (GPa) alongside experimental and theoretical results.

Comp	*XC*	*C*_11_	*C*_12_	*C*_13_	*C*_33_	*C*_44_	Refs.
Ti_3_AlC_2_	PBE	277	93	70	242	114	This work
	Exp	361	75	70	299	124	^47^
	Others	353	75	69	296	119	^47^
Ti_3_SiC_2_	PBE	326	98.3	115	317	143	This work
	Exp	365	125	120	375	122	^47^
	Others	366	94	100	352	153	^47^

A stable hexagonal crystal must satisfy the following Born-Huang stability criteria^46^;

2 $${C}_{11}>0; {C}_{11}-{C}_{12}>0; {C}_{44}>0; \left({C}_{11}+  C_{12}\right){C}_{33}-{2C}_{13}^{2}>0$$

Table 2 demonstrates that the computed results of the independent elastic constants for Ti_3_SiC_2_ and Ti_3_AlC_2_ MAX phase compounds satisfy the mechanical stability criteria which signify that all the compounds are mechanically stable. It is also well known that elastic constants *C*_11_, and *C*_33_ shows linear compression resistances along *a* and *c* directions, respectively, whereas *C*_12_, *C*_13,_ and *C*_44_ are related to the shape elasticity. Consistent with Table 2, the value of C_11_ is higher than C_33_ for both Ti_3_SiC_2_ and Ti_3_AlC_2_ compounds which agrees well with literature results.

From the computed elastic constants, several polycrystalline elastic moduli comprising, Bulk, Shear*,* Young moduli, and Poisson’s ratio were evaluated using Voigt^48^, Reuss^49^, and Hill^50^ approximations. It is assumed that, in the Voigt scheme, the strain is uniform all along the polycrystalline materials aggregating to external strain. By following this approach, for the hexagonal lattices, the Voigt shear modulus (G_V_) and Reuss shear modulus (G_R_) are expressed as:

3 $${G}_{V}= \frac{1}{15}\left\{2{C}_{11}-{C}_{12}+{C}_{33}-{2C}_{13}\right\}+\frac{1}{5}\left\{2{C}_{44}+\frac{1}{2}\left( {C}_{11}-{C}_{12}\right)\right\}$$

4 $${G}_{R}=\frac{5}{2}\left\{\frac{\left[\left[\left({C}_{11}+{C}_{12}\right){C}_{33}-2{C}_{13}^{2}\right]{C}_{44}{C}_{66}\right]}{\left[3{B}_{V}{C}_{44}{C}_{66}+\left[\left({C}_{11}+{C}_{12}\right){C}_{33}-2{C}_{13}^{2}\right]\left({C}_{44}+{C}_{66}\right)\right]}\right\}$$

And Voigt bulk modulus (*B*_*V*_), Reuss bulk modulus (*B*_*R*_) by:

5 $${B}_{V}= \frac{1}{9}\left\{2\left({C}_{11}+ {C}_{12}\right)+{C}_{33}+4{C}_{13}\right\}$$

6 $${B}_{R}= \frac{\left({C}_{11}+{C}_{12}\right){C}_{13}-2{C}_{13}^{2}}{{C}_{11}+ {C}_{12}+2{C}_{33}+4{C}_{13}}$$

Hill showed that Voigt/Reuss averages give upper and lower bounds, and therefore, proposed that real effective moduli can be approximated by the arithmetic mean of the two bounds^51^. Thus, using Hill’s approximations

7 $$B=\frac{1}{2}\left({B}_{R}+{B}_{V}\right),\quad G=\frac{1}{2}\left({G}_{R}+{G}_{V}\right)$$

We have also computed $Y$, and $\eta$, which are commonly evaluated for polycrystalline materials to study their hardness. Both $Y$ and $\eta$ are defined by the following expressions as;

8 $$Y=\frac{9BG}{3B+G} , \quad\eta =\frac{3B-2G}{2\left(3B+G\right)}$$

The computed Bulk modulus, Young’s modulus, Shear moduli, and Poisson’s ratio of both Ti_3_SiC_2_ and Ti_3_AlC_2_ as defined in Eqs. (3)–(8) are listed in Table 3. The calculated values for the bulk modulus of Ti_3_SiC_2_ and Ti_3_AlC_2_ are 139 GPa and 182 GPa respectively. These values agree well with the reported value by Gray et al.^47^, with less than 13% and 7% deviation respectively for Ti_3_AlC_2_ and Ti_3_SiC_2_. Moreover, our results for Shear modulus of 87 GPa for Ti_3_AlC_2_ although are lower than the reported experimental value in Table 3, the results of Ti_3_SiC_2_ of 121 GPa are in good agreement with the reported value. From comparing Tables 1 and 3, it can be seen that the calculated value of *B* obtained from the single crystal elastic constants summarized in Table 3 has approximately the same value as the one obtained from the data fitting in the Murnaghan’s equation of state (Table 1). This indicates the accuracy and reliability of our computed elastic constants for both Ti_3_SiC_2_ and Ti_3_AlC_2_ MAX phase compounds.

**Table 3:** Computed bulk modulus *B* (GPa), Young modulus *Y*(GPa), shear modulus *G* (GPa), Poisson’s ratio *η,* compressibility *B*^−1^ (GPa)^−1^, Pugh’s ratio (*B/G*), Cauchy pressure ${C}_{c} \mathrm{(GPa)},$ anisotropic factor-*A* and Vicker’s hardness *H*_v_*.*

Comp	*B*	*Y*	*G*	η	*B*^−1^	*B*/*G*	${C}_{c}$	*A*	*H*_V_	Refs.
Ti_3_AlC_2_	139	215	87	0.24	0.007	1.60	− 44	1.203	40.28	This work
	161.2	321	132	0.178	0.006	1.22	− 50	0.971	36.88	^18,47^
Ti_3_SiC_2_	189	297	121	0.23	0.005	1.56	− 28	1.398	46.75	This work
	203.9	307	123	0.248	0.005	1.66	− 53	1.202	25.53	^18,47^

Following the Pugh ratio, $B/G$ shows the brittle or ductile character of materials. Pugh’s critical value is 1.75. The calculated ratio *B/G* for Ti_3_AlC_2_ and Ti_3_SiC_2_ are 1.60 and 1.56, respectively, which are less than Pugh’s critical value. As such, these compounds have a brittle feature which agreed well with the result given in Table 3^52^. Cauchy relation defined as: ${C}_{c}={C}_{13}-{C}_{44}$, is another parameter signifying ductility or brittleness of a material. Positive values of ${C}_{c}$ shows ductility otherwise the material is brittle^53^. The evaluated ${C}_{c}$ of the ternaries are -44 and -28 GPa respectively. From these values, one can conclude that the studied materials are brittle in nature which confirmed the Pugh’s result. Consequently, the brittle nature of Ti_3_AlC_2_ and Ti_3_SiC_2_ can be related to their ceramic character.

Young’s modulus (*Y*) measures the stiffness of a material. The higher the *Y*, the stiffer a material is. Our result presented in Table 3 shows that there is good agreement with the reported values of 215 GPa and 297 GPa for Ti_3_AlC_2_ and Ti_3_SiC_2,_ respectively. Information about the bonding forces can be obtained via Poisson’s ratio (*η*). The$\left(\eta \right)$ for Ti_3_AlC_2_ and Ti_3_SiC_2_ are 0.24 and 0.23 respectively, which shows the interatomic forces within studied materials are central since upper and lower limits of the Poisson’s ratio is $0.5$ and $0.25$ respectively, and the calculated values fall within the two limits. Our results are closer to the experimental value of 0.178 for Ti_3_AlC_2_ and 0.248 for Ti_3_SiC_2_^52^. We have further calculated the Vickers’ hardness *H*_v_^54^ of studied compounds. Vickers’s hardness is another key mechanical property of solids that explains stability, which is predicted using Eq. (9). It is reported that solids with Vickers hardness *H*_V_ > 40 GPa^38^ are graded as super hard solids. The calculated *H*_v_ of Ti_3_AlC_2_ and Ti_3_SiC_2_ MAX phase compounds are 40.28 GPa, and 46.75 GPa respectively (Table 3). Therefore, these crystals, have an excellent ability to withstand dents or scratches.

9 $${H}_{V}=0.92{\left(\frac{B}{G}\right)}^{1.3137}{G}^{0.708}$$

## Method

Ab initio calculations were used to investigate the elastic, and electronic properties of Ti_3_SiC_2_ and Ti_3_AlC_2_ using PW-PP as implemented in Quantum Espresso^55^. Generalized gradient approximation (GGA) parametrized by Perdew-Burke-Ernzerhof (PBE) is used to treat exchange and correlation (XC) energy^56^. The core ion and valence electrons interactions were described using ultrasoft-pseudopotentials (UPP). A 600 Ry kinetic energy cut-off of the plane wave is used in the calculations. The electronic configurations: 3s^2^, 4s^2^, 3p^6^, 3d^2^ for Ti, 3s^2^, 3p^2^ for Si, 3p^1^, 3s^2^ for Al and 2s^2^, 2p^2^ for C were considered for the valence electrons. For the Brillouin zone (BZ) integration, 12 × 12 × 12 k-points mesh was generated using the Monkhorst–Pack scheme^57^. These parameters were found to be adequate to converge total energies up to 10^–8^ eV. Both studied materials were fully relaxed in terms of cell parameters and atomic positions. Analysis of independent elastic constant $({C}_{ij})$ were performed using thermo_pw^45^. ${C}_{ij}$ delineates response of materials to macroscopic stress. In computing elastic constants, a small strain, *e* is applied to a material and the variation of total energy per volume, *U* of the material is obtained^58^:

10 $$U=\frac{\Delta E}{{V}_{0}}=\frac{1}{2}\sum_{i}^{n}\sum_{j}^{m}{C}_{ij}{e}_{i}{e}_{j}$$

where ${V}_{0}$ and $\Delta E$ represent the equilibrium volume and the difference between the initial and deformed total energy of the system respectively. The hexagonal Ti_3_SiC_2_ and Ti_3_AlC_2_ MAX phase compounds are characterized by five independent elastic constants which include ${C}_{11}$, ${C}_{12}$, ${C}_{13}$,${C}_{33}$ and ${C}_{44}$. Therefore, the elastic matrix of the hexagonal system is written as^59,60^;

11 $${C}_{ij}=\left[\begin{array}{cccccc}{C}_{11}& {C}_{12}& {C}_{13}& .& .& .\\ .& {C}_{11}& {C}_{13}& .& .& .\\ .& .& {C}_{33}& .& .& .\\ .& .& .& {C}_{44}& .& .\\ .& .& .& .& {C}_{44}& .\\ .& .& .& .& .& .\end{array}\right]$$

## Conclusion

In this work, the structural stability, electronic, and mechanical properties were investigated using ab-initio calculations. The complete set of independent elastic constants *C*_ij,_ shear modulus, bulk modulus, Poisson’s ratio*,* and Young’s modulus were calculated. Our results showed that the studied ternaries are mechanically stable and are super hard materials with Vicker’s hardness as large as 46.75 GPa and 40.28 GPa for Ti_3_SiC_2_ and Ti_3_AlC_2_ respectively. The investigated electronic band structures, TDOS, and PDOS showed the metallic behavior of these compounds. In Ti_3_SiC_2_, the top of the VB and bottom of the CB were found to be dominated by the Si-3p, C-2p, and Ti-3d energy states while for the Ti_3_AlC_2_ the top and bottom of VB and CB were respectively found to be shaped by Al-3p, C-2p, and Ti-3d orbitals. We expect that our findings will provide suitable guidance for experimental and theoretical studies on these interesting MAX phases.
